# Peanut Consumption in Malawi: An Opportunity for Innovation

**DOI:** 10.3390/foods7070112

**Published:** 2018-07-14

**Authors:** Aggrey P. Gama, Koushik Adhikari, David A. Hoisington

**Affiliations:** 1 Department of Food Science and Technology, University of Georgia, 1109 Experiment St, Griffin, GA 30223, USA; aggrey.gama25@uga.edu; 2 Department of Food Science and Technology, Lilongwe University of Agriculture and Natural Resources, P.O. Box 219, Lilongwe, Malawi; 3 Peanut Innovation Lab, University of Georgia, 217 Hoke Smith Building, Athens, GA 30602, USA; davehois@uga.edu

**Keywords:** peanut, peanut consumption, Malawian consumer, consumer behavior, logistic models

## Abstract

Peanuts are a valuable source of nutrients, but peanut consumption patterns, consumption frequencies, and the factors influencing peanut consumption in Malawi are not known. This study surveyed consumers to fill this knowledge gap and to assess Malawian consumers’ readiness to try new food products. Out of the 489 respondents surveyed, all but three consumed peanuts (in any form). The majority (70.4%) consumed peanuts at least three times in a week. Chi-square test showed that demographic and socioeconomic variables had significant effects (*p* < 0.05) on peanut product preferences, the frequency of peanut consumption, and readiness to try new foods. For instance, women mostly preferred peanut flour compared to men, and peanut butter was the most preferred form for younger consumers. Logistic regression analysis showed that consumers with high school education or below were 2.35 times more likely to eat peanuts more often than consumers with post high school education. Among the participants that were ready to try new foods (54%), men and those with post high school education were 1.90 and 2.74 times more likely to try new foods than their respective counterparts. In general, the diversity of peanut products on the Malawian market is limited, and socioeconomic restrictions override consumer preferences. Therefore, future peanut-based food products innovations should explore ways to overcome such restrictions.

## 1. Introduction

Prevalence of malnutrition, especially under-nutrition, is high in Sub-Saharan Africa (SSA) as compared to the developed countries. Between 1990 and 2014, there was a 24% overall increase in the number of undernourished people in SSA. As a way of addressing undernutrition, governments in the region are implementing initiatives designed to increase production of and access to nutritious foods [1]. For instance, the Malawian government is promoting dietary diversification by supporting production and consumption of underutilized yet nutritious foods like peanuts [2]. 

Peanuts are a valuable source of proteins, fats, vitamins, and minerals for human and animal nutrition. Peanut kernels, on average, contain 48% fat, 26% protein, 17% carbohydrates, 2% fiber, 2% ash, and 1% of vitamins and minerals which include vitamin E, niacin, folate, manganese, magnesium, and phosphorous [3]. Peanuts also contain bioactive substances such as flavonoids, resveratrol, and phytosterols, which have been associated with decreased risk of coronary heart disease (CHD) and reduced cancer risk [4,5]. Many studies have been done on the health benefits derived from peanut consumption. For instance, Griel et al. [6] found that peanut users have a higher intake of micronutrients, lower intake of saturated fat and cholesterol, and lower body mass index (BMI), despite peanuts being energy-dense. The Food and Agriculture Organization (FAO) [7] suggested that a handful (~30 g) of peanuts a day could be enough to address most malnutrition cases in developing countries. Unfortunately, peanut consumption in Malawi is quite low (~13 g/day.) although production is relatively high [8]. The reasons behind the low peanut consumption are not known because peanut consumption patterns, consumption frequencies, and the factors influencing peanut consumption in Malawi have not been determined. 

Although making food choices is a complex process, it is known that consumers choose foods or food products that meet their needs and wants [9,10]. The consumer needs and wants ultimately influence food choices and consumption patterns. Unfortunately, identification of the motivating factors that would make people consume more nutritious foods like peanuts is often overlooked. Nutrition interventions aimed at promoting consumption of particular foods in SSA have been mostly done considering only health benefits, although studies, especially in developed countries, have shown that health is just one of the several factors that influence food choices [11,12,13,14]. Furthermore, the interventions are not tailored for different target groups although evidence shows that food consumption patterns, preferences, and dominant food choice motives vary among nationalities, cultures and socio-demographic profiles [11,13,14,15,16].

Given the above, a consumer survey was conducted to identify the peanut consumption patterns, consumption frequencies, and the factors that influence consumption of peanuts in Malawi. Furthermore, the effect of consumers’ demographic and socioeconomic profiles on peanut consumption, peanut preferences, and readiness of Malawian consumers to try new foods was also investigated. Although the results are based on a survey from Malawi, they could also be useful to other nutritionists, food scientists, policymakers, food companies, and food marketers in Sub-Saharan Africa, especially countries in southern Africa with relatively similar dietary patterns to Malawi.

## 2. Materials and Methods 

### 2.1. Procedure and Subjects

A consumer survey was conducted in three major cities (Lilongwe, Blantyre, and Mzuzu) of Malawi. Invitations to participants were sent through flyers, posters, and billboards where the aims of the study and the inclusion criteria, among others, were briefly described. In each city, participants came to one designated place and completed a questionnaire in the presence of the researchers. There were 169 participants recruited in Lilongwe (Centre), 162 in Blantyre (South) and 158 in Mzuzu (North). The participants were diverse in their gender, age, education level, occupation, and monthly incomes. Only Malawian adults (aged 18 or above) who make independent food choice decisions participate. All human subjects ethical procedures were followed as approved by the University of Georgia’s Institutional Review Board (IRB Approval Number: STUDY00004112).

### 2.2. Data Collection Tool

The questionnaire was provided in English, the official language, and Chichewa, the main local language in Malawi. The questionnaire was translated into the local language and back-translated into English by two independent translators for accuracy and good language equivalence. The questionnaire had three sections. The first section was on peanut consumption and preferences followed by a section on food neophobia. In the first section, participants were asked to mention all the peanut products that they consumed, and how often they consumed the peanut products. Then, they were asked to mention their most preferred peanut form or product and to give reasons for their choice. In the food neophobia section, participants were asked if they would try new (unfamiliar) foods or food products. For those who indicated that they would not try new foods, their reasons for fearing new foods and what could compel them, if possible, to try new foods were solicited through open-ended questions. The last section was about the participants’ demographic and socioeconomic information. Data on gender, age, highest education level, occupation, and monthly incomes were collected.

### 2.3. Data Analysis

Frequency distributions were generated, and the differences in proportions were assessed using χ^2^ test or Fisher’s exact test where theoretical cell counts were less than 5. Likelihood of a consumer to like a particular peanut product, based on gender, age, education level, occupation, and monthly income, was determined using binary logistic regression analysis. Regression coefficients were estimated using maximum likelihood estimation and are presented with odds ratios (ORs) and Wald χ^2^-statistics. The ORs are point estimates obtained by exponentiating the estimate. The OR indicates the likely change in the probability of the response factor if there is a one unit increase in the level of the predictor variable when all other variables are held constant. When the OR is equal to 1, there is no effect of the unit variation of the predictor on the response factor. A large deviation of the OR values from one signifies a greater effect on change in the occurrence probability of the response factor and vice versa. The Wald χ^2^-statistic is the test statistic for the hypothesis test that an individual predictor’s estimate (regression coefficient) is zero given the rest of the predictors are in the model. The Wald χ^2^-statistic is the squared ratio of the estimate to the standard error of the respective predictor [17]. The logistic model used in this study is represented as follows:

(1) $$\;P\left(Y_{i}=1│X\right)=\;\frac{1}{1+\;e^{-(\beta_{i}+\sum_{i=1}^{n}\beta_{i}X_{i})}}\;$$

The binary dependent response (*Y*_i_) for participant *i* has a value of 1 if the participant likes the specified peanut product, otherwise, it has a value of 0. Vectors of the exploratory variables are represented by *X*_i_ while the intercept and variable coefficients are represented by *β_0_* and *β_i_* (*i* = 1, 2 …n), respectively. For modeling purposes only, age was categorized as young (<30 years) and old (≥30 years), education level as higher level (post high school education) and lower level (high school and below), monthly income as lower earners (low income) and higher earners (medium and high income), and occupation was categorized as ‘full-time job’ and other occupations (self-employed, menial jobs, students, and none). A similar analysis was done to estimate the likelihood that a consumer will try new foods or not and whether a consumer is likely to eat peanuts or peanuts products more often (at least thrice a week) or less often (at most once a week). All statistical analyses were done in XLSTAT 2017 (Addinsoft, New York, NY, USA). 

## 3. Results

### 3.1. Study Participants

The demographic and socioeconomic information of the participants is shown in Table 1. Of the 489 participants, there were more men (68%) than women, and most of the participants were aged between 21 and 39 (73%).

### 3.2. Peanut Consumption and Preference

**Figure 1:**
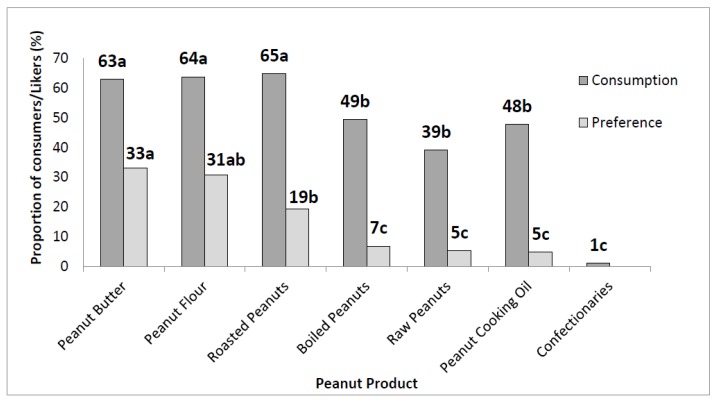
Peanut products and their respective consumption and preference frequencies. Unlike for preference, the sum of consumption frequencies is more than 100% because multiple responses were allowed. Values followed by common letters (a, b, or c) indicate no significant difference (*p* > 0.05).

Out of the 489 respondents surveyed, all but three consumed peanuts, peanut products, or both. The three who did not eat peanuts and peanut products indicated that this was because of allergies. The most frequently mentioned forms of peanut consumption were roasted peanuts (65%), peanut flour (64%), and peanut butter (63%), as shown in Figure 1. However, the most preferred forms were peanut butter (33%), peanut flour (31%), and roasted peanuts (19%). A summary of peanut product preference by gender, age, education level, occupation, and monthly income is shown in Table 1.

**Figure 2:**
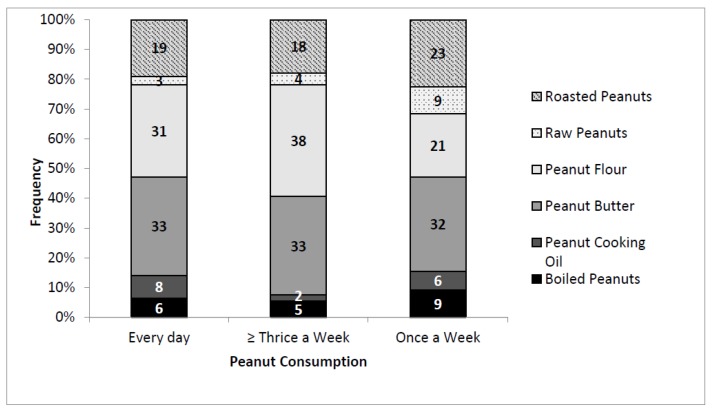
Peanut consumption rate related to consumer preference for the various peanut products. Frequency is the proportion of respondents categorized based on their most preferred peanut products.

The majority (70.4%) consumed peanuts (in any form) at least three times in a week. Peanut flour and peanut butter were the most preferred peanut forms for those that consume peanuts at least three times a week or more (Figure 2). Consumers who ate peanuts at least once a week mostly liked peanut butter and roasted peanuts. A summary of peanut consumption frequency by gender, age, education level, occupation, and monthly income is shown in Table 1.

### 3.3. Drivers of Peanut Preferences

**Figure 3:**
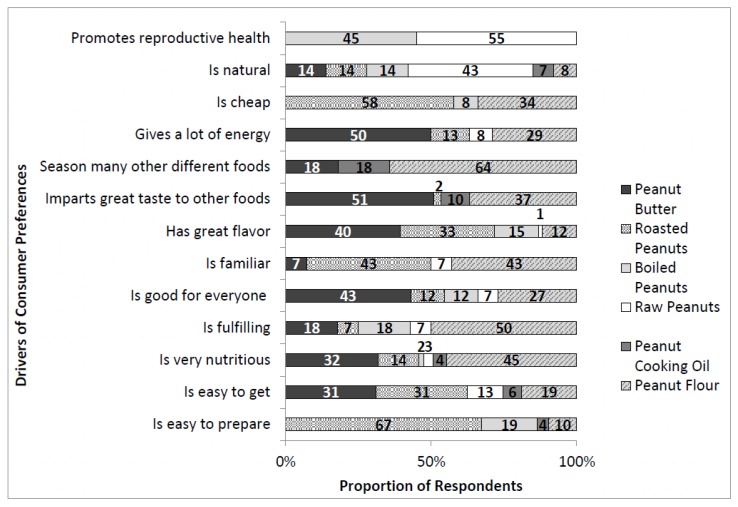
Reasons behind consumer preferences for the various peanut products. Frequency is the proportion of respondents who gave a reason, categorized based on their most preferred peanut products.

All of the three most preferred peanut products were considered to be very nutritious (Figure 3). Distinctively, peanut flour preference was mainly due to its versatility since it can be used to season many other foods. The preference for roasted peanuts was primarily due to price and preparation convenience because it is cheap and easy to prepare. Peanut butter was the most preferred form of peanut consumption due to its sensory appeal and the fact that it is energy-dense. Notably, preference for both raw and boiled peanuts was mainly due to their perceived ability to enhance reproductive health.

### 3.4. Readiness to Try New Foods 

**Figure 4:**
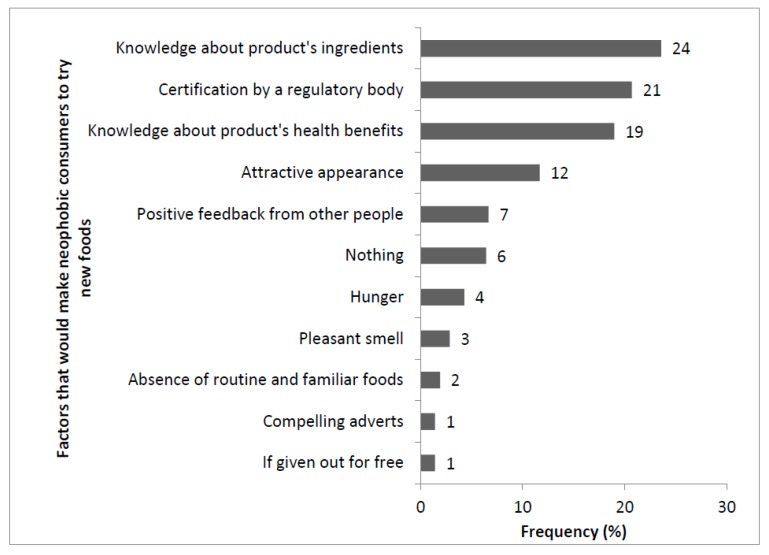
Compelling reasons for ignoring fear for new foods as indicated by the neophobic consumers (*n* = 225).

There was a significant difference between the proportions of neophobic and neophilic respondents (*p* < 0.05). Fifty-four percent of the respondents indicated that they were willing to try new food products for the first time. For participants that were ready to try new foods, a frequency summary by gender, age, education level, occupation, and monthly income is shown in Table 1. The consumers who were not ready to try new foods (neophobic consumers) had concerns about safety in general (81%), nature of the ingredients (13%), and allergens (6%). As a result, safety assurance and knowledge about the ingredients were the most frequently mentioned factors that would compel them to try new foods despite their food neophobic dispositions (Figure 4). However, a few consumers (6%) indicated that nothing could compel them to try new foods.

### 3.5. Effect of Demographic and Socioeconomic Variables on Peanut Preference 

Differences were noted in the associations between preferences for the various peanut products and the demographic and socioeconomic variables (Table 2). Gender, age, education level, occupation, and monthly income, respectively, had significant associations with consumer preference for peanut cooking oil (*p* < 0.05). Men were 2.56 times more likely to like peanut cooking oil than women when all other factors were held constant. However, younger consumers, irrespective of gender, were 1.63 times more likely to like peanut cooking oil than older consumers. Likewise, consumers with high school education or below, those with full-time jobs, and consumers with lower monthly incomes, respectively, were 2.05, 2.04, and 4.33 times more likely to prefer peanut cooking oil than their counterparts.

Preference for raw peanuts was significantly (*p* < 0.05) influenced by each of the assessed demographic and socioeconomic variables except occupation. Men were 3.80 times more likely to prefer raw peanuts than women. Likewise, older consumers, those with post high school education, and consumers with lower monthly incomes, respectively, were 2.17, 2.99, and 2.30 times more likely to like raw peanuts than their counterparts. 

Gender, age, and education level, respectively, had a significant influence on preference for peanut flour (*p* < 0.05). Women, those with high school education or below, and older consumers, respectively, were 1.86, 1.70, and 1.71 times more likely, respectively, to prefer peanut flour compared to their counterparts.

Preference for boiled peanuts was significantly influenced by education level and monthly incomes (*p* < 0.05). Consumers with post high school education were 1.56 times more likely to like boiled peanuts than those in lower education level category. Likewise, consumers with higher monthly incomes were 2.05 times more likely to prefer boiled peanuts compared to consumers with lower monthly incomes.

Gender and occupation, respectively, had a significant influence on preference for roasted peanuts (*p* < 0.05). Men and those that had full-time jobs were 2.15 and 1.48 times more likely to like roasted peanuts compared to their respective counterparts. 

Finally, preference for peanut butter was significantly influenced by age and education level, respectively (*p* < 0.05). Consumers with post high education and those that were younger were 1.54 and 1.70 times more likely to prefer peanut butter than their respective counterparts.

### 3.6. Effect of Demographic and Socioeconomic Variables on Peanut Consumption Frequency

As shown in Table 3, peanut consumption frequency, irrespective of peanut form, was significantly influenced by education level only (*p* < 0.05). Consumers with high school education or below were 2.35 times more likely to eat peanuts (in any form) more often than consumers in the high education level category.

### 3.7. Effect of Demographic and Socioeconomic Variables on Readiness to Try New Foods

Gender, education level, and occupation, respectively, had a significant effect on readiness to try new foods as shown in Table 3 (*p* < 0.05). Men were 1.90 times more likely to try new foods than women. Likewise, consumers with post high school education and those with full-time jobs were 2.74 and 2.02 times more likely, respectively, to try new foods compared to their counterparts. 

## 4. Discussion

Based on the findings, it is evident that the diversity of peanut products in Malawi is limited. Unlike in other countries where peanuts are eaten in many different forms, peanuts in Malawi are consumed in a few typical customary forms such as peanut flour, raw, boiled, and roasted peanuts. As expected, these are peanut products that that are easy to prepare within households either for consumption or for vending. George Washington Carver is credited for his significant contribution to the development of peanut products. His research resulted in the development of 300 products and 105 ways of using peanuts in soups, puree, bread, candy, cheeses, coffee, cookies, cakes, puddings, ice creams, cutlets, patties, sausages, omelets, macaroni, stuffing, wafers, bars, and doughnuts, among others [18,19]. The absence of most of these peanut-based products on the Malawian market is an opportunity for peanut product diversification. The limited diversity of peanut products on the Malawian market is likely one of the factors contributing to the low peanut consumption.

The mismatch between consumption and preferences is also an opportunity for innovation. One would expect a food product that is mostly preferred to be the most consumed product as well. However, as found in this study, peanut butter which was the most preferred peanut product was third when the products were ranked based on consumption. Consumers indicated that they preferred peanut products like peanut flour, boiled peanuts, and roasted peanuts because they were cheap and easy to prepare, but no one mentioned these as reasons for his or her peanut butter preference. Deductively, it is likely that peanut butter consumption is hindered by its relatively high price and inconvenient preparation process. Therefore, new peanut-based products that could have the desired sensory properties like peanut butter, affordable, and convenient are likely to succeed on the Malawian market.

The significant effect of demographic and socioeconomic variables on peanut products preference provides useful insights into Malawian consumers’ characteristics and their peanut consumption patterns. The significant demographic and socioeconomic effects can also serve as a basis for classifying the peanut products in Malawi. Peanut flour could be classified as a product for women, older consumers, and those in the low education level category (high school education and below). Roasted peanuts are for men and those with full-time jobs. Peanut butter is for younger consumers and those with post high school education. Boiled peanuts are for consumers with post high school education and those with higher monthly incomes. Raw peanuts are for men, older consumers, those with post high school education, and consumers with lower monthly incomes. Peanut cooking oil seemed to be preferred by men in general, younger consumers, those in the low education level category (high school education and below), consumers with full-time jobs, and those with lower monthly incomes. This might be related to flavor of foods cooked or fried in peanut oil rather than just preference for the oil itself.

The effect of demographic and socioeconomic variables on peanut consumption has also been found in studies done in other countries. For instance, just like in Malawi, men were also found to be the dominant consumers of raw peanuts in Ghana and two southern states of the USA, respectively [20,21]. Men consider peanuts, especially raw peanuts, as an aphrodisiac [21]. Therefore, it is not surprising that enhancement of reproductive health was the most frequently mentioned reason for preferring raw and boiled peanuts. Peanuts are a rich natural source of L-arginine. This amino acid helps improve sexual function by relaxing blood vessels [22]. 

The reasons behind peanut preferences in Malawi are related to health (nutritious, good for reproductive health, provides energy), convenience (easy to prepare, easy to get, good for everyone), familiarity (is familiar), sensory appeal (great flavor, fulfilling, imparts great taste to other foods), price (is cheap), natural content (is natural), and versatility (can be used to season many other foods). Other studies also found that health, convenience, familiarity, price, sensory appeal, and natural content of foods as factors that influence food choices of consumers in general [11,12,13,14,23,24]. Therefore, only versatility is the unique factor identified in this study. Peanut flour was considered to be highly versatile among all the peanut products identified in this study. In Malawi, peanut flour is added to many other different foods such as vegetables, fish, corn flour porridge, sweet potatoes, plantains, rice, and corn grits. It can also be used to make peanut sauce (Thendo) by mixing the peanut flour with other ingredients such as tomatoes. 

The significant effect of demographic and socioeconomic factors on peanut consumption frequency and readiness to try new foods provides further insights into the characteristics of Malawian consumers. The findings have shown that Malawian consumers with post high school education eat peanut products less often compared to those in the low education level category. Whether the peanuts are replaced with other equally nutritious foods or snacks is not known. Consumers with post high school education were also more ready to try new foods than those in the low education level category. Likewise, men and those with full-time jobs were more ready to try new foods compared to their respective counterparts. Based on this study, the neophilic consumers have high purchasing power since they have higher monthly incomes compared to their respective counterparts. Therefore, targeting them with new commercial food products which meet their needs and wants could be feasible. Food neophobic individuals consider novel or unfamiliar foods as a threat, and therefore they react negatively to these products [25]. Food neophobia restricts marketability of new food products, is a possible barrier to a balanced diet, and can restrict change towards a healthier diet [26,27,28]. Therefore, food product marketing strategies should be devised to target this segment of neophobic consumers when promoting new food products. Apart from the few consumers, those for which nothing can compel them to try new foods, most of the food neophobic consumers are just being cautious. Addressing their fears by providing safety assurance and declaring the product’s ingredients, among other actions, could yield positive results. Veeck [25] noted that food neophobia could be described as an individual and a social trait. Therefore, as found in this study, a referral from a trusted friend or close family member may persuade a food neophobic person to sample an unfamiliar food product. However, further studies should be done to determine the effectiveness of the factors, identified in this study, which could compel a neophobic individual to try new foods. Unlike the approach used in this study, such further studies should use the validated tool developed by Pliner and Hobden [29] for measuring food neophobia to maximize the reliability of the findings.

## 5. Conclusions

This study has provided useful information on peanut consumption in Malawi, the reasons for peanut product preferences, and the differences in consumer preferences for the various peanut forms based on demographic and socioeconomic factors. The limited diversity of peanut products on the Malawian market is an opportunity for innovation. Given that socioeconomic restrictions override consumer preferences, future peanut-based food products innovations should, therefore, explore ways to strike a balance between price and the other food choice motives. Furthermore, proper marketing strategies should be developed to target neophobic consumers.

In this study, the sample size was statistically adequate, and the socioeconomic profiles of the participants provided a relatively good representation of Malawian consumers. However, the authors acknowledge the limitation that the sample was not demographically balanced when compared to the national population distribution. Therefore, the findings are suggestive of the likely national trend rather than being conclusive. However, this is the first study of its kind in Malawi; therefore, the results have provided good insights into Malawian consumers’ peanut consumption patterns, preferences, and their readiness to try new foods. Future studies can build on the findings of this study to further explore issues related to peanut consumption and food neophobia in Malawi, preferably using a purposive sampling plan to balance the demographic composition of the participants. 

## Figures and Tables

**Table 1:** Summary of demographic, socioeconomic information of the respondents, and their respective frequencies for peanut product consumption, preference, and readiness to try new foods.

Variable	Total Respondents % (*n* = 489)	Ready to Try New Foods % (*n* = 264)	Consumption Frequency (%)			Preference Frequency (%)					
			Everyday (*n* = 143)	≥3 Times/Week (*n* = 201)	Once/Week (*n* = 144)	PB (*n* = 161)	PF (*n* = 151)	RP (*n* = 95)	BP (*n* = 33)	RwP (*n* = 26)	PCO (*n* = 23)
Gender											
Male	68 (333)	73 (194)	64 (91)	69 (139)	70 (101)	65 (104)	58 (88)	79 (75)	64 (21)	88 (23)	87 (20)
Female	32 (156)	27 (70)	36 (52)	31 (62)	30 (43)	35 (57)	42 (63)	21 (20)	36 (12)	12 (3)	13 (3)
Age											
18–20 years	10 (49)	10 (27)	15 (22)	6 (12)	12 (17)	16 (25)	10 (15)	6 (6)	3 (1)	8 (2)	9 (2)
21–29 years	41 (200)	45 (117)	37 (53)	44 (89)	39 (56)	45 (73)	33 (49)	43 (41)	49 (16)	30 (8)	52 (12)
30–39 years	32 (156)	29 (77)	30 (43)	35 (70)	31 (45)	29 (46)	36 (55)	32 (30)	30 (6)	46 (12)	22 (5)
40–49 years	12 (59)	12 (32)	12 (17)	10 (19)	14 (20)	8 (13)	12 (18)	15 (14)	18 (6)	8 (2)	13 (3)
≥ 50 years	5 (25)	4 (11)	6 (8)	5 (11)	4 (6)	2 (4)	9 (14)	4 (4)	0 (0)	8 (2)	4 (1)
Highest Education Level											
None	1 (5)	1 (2)	1 (1)	1 (3)	1 (1)	1 (1)	1 (2)	2 (2)	0 (0)	0 (0)	0 (0)
Primary (Pre high school)	12 (59)	6 (16)	19 (27)	10 (20)	6 (9)	6 (10)	20 (30)	13 (12)	6 (2)	8 (2)	0 (0)
Secondary (High school)	51 (249)	45 (120)	59 (85)	53 (106)	41 (59)	50 (80)	52 (79)	50 (48)	42 (19)	38 (10)	83 (19)
Tertiary (Post high school)	36 (176)	48 (126)	21 (30)	36 (72)	52 (75)	43 (70)	26 (40)	35 (33)	52 (17)	54 (14)	17 (4)
Occupation											
Full-time job	40 (196)	48 (127)	35 (50)	40 (80)	46 (66)	38 (61)	36 (55)	46 (44)	43 (14)	46 (12)	47 (11)
Self-Employment	27 (132)	25 (67)	34 (48)	27 (54)	22 (31)	24 (39)	28 (42)	32 (30)	30 (10)	27 (7)	22 (5)
Menial Job	14 (68)	8 (20)	11 (16)	17 (35)	12 (18)	12 (19)	22 (33)	12 (11)	9 (3)	4 (1)	9 (2)
Student	14 (68)	17 (44)	14 (20)	10 (21)	18 (26)	22 (35)	8 (12)	8 (8)	15 (5)	19 (5)	9 (2)
None	5 (25)	2 (6)	6 (9)	6 (11)	2 (3)	4 (7)	6 (9)	2 (2)	3 (1)	4 (1)	13 (3)
Monthly Income (MK) ^a^											
<100,000 (Low)	78 (381)	75 (197)	81 (116)	80 (161)	71 (102)	78 (126)	80 (121)	73 (69)	64 (21)	77 (20)	96 (22)
100,000–499,999 (Medium)	18 (88)	21 (55)	14 (20)	16 (33)	24 (34)	18 (28)	17 (26)	19 (18)	33 (11)	19 (5)	0 (0)
≥500,000 (High)	4 (20)	4 (12)	5 (7)	5 (7)	5 (8)	4 (7)	3 (4)	8 (8)	3 (1)	4 (1)	4 (1)

^a^ 1 US Dollar ($) = 700 Malawian Kwacha (MK); Numbers in parenthesis correspond to the number of observation. PB = peanut butter, PF = peanut flour, RP = roasted peanuts, BP = boiled peanuts, RwP = raw peanuts, PCO = peanut cooking oil.

**Table 2:** Logistic regression estimates for demographic and socioeconomic variables influencing peanut product preference.

Predictor	Peanut Butter		Peanut Flour		Roasted Peanuts		Boiled Peanuts		Raw Peanuts		Peanut Cooking Oil	
	Odds Ratio (CI)	χ^2^ *p* > χ^2^	Odds Ratio (CI)	χ^2^ *p* > χ^2^	Odds Ratio (CI)	χ^2^ *p* > χ^2^	Odds Ratio (CI)	χ^2^ *p* > χ^2^	Odds Ratio (CI)	χ^2^ *p* > χ^2^	Odds Ratio (CI)	χ^2^ *p* > χ^2^
Gender												
Male vs. Female	1.30 (0.88, 1.91)	1.755 0.185	1.86 (1.27, 2.74)	9.937 0.002	2.15 (1.42, 3.24)	13.184 0.000	1.15 (0.78, 1.70)	0.510 0.475	3.80 (2.31, 6.24)	27.817 <0.0001	2.56 (1.57, 4.17)	14.176 0.000
Age												
<30 years vs. ≥30 years.	1.70 (1.15, 2.51)	6.984 0.008	1.71 (1.15, 2.52)	7.183 0.007	1.09 (0.74, 1.62)	0.205 0.651	1.25 (0.82, 1.91)	1.115 0.291	2.17 (1.43, 3.29)	13.352 0.000	1.63 (1.06, 2.51)	5.005 0.025
Education level												
High school and below vs. Post high school	1.54 (1.03, 2.28)	4.493 0.034	1.70 (1.12, 2.59)	6.209 0.013	1.34 (0.89, 2.02)	1.927 0.165	1.56 (1.03, 2.37)	4.344 0.037	2.99 (1.93, 4.63)	24.153 <0.0001	2.05 (1.26, 3.34)	8.394 0.004
Occupation												
Full-time employment vs. Other occupations	1.18 (0.80, 1.74)	0.665 0.415	1.17 (0.79, 1.74)	0.621 0.431	1.48 (1.00, 2.18)	3.928 0.047	1.24 (0.83, 1.86)	1.088 0.297	1.27 (0.85, 1.87)	1.370 0.242	2.04 (1.34, 3.09)	11.133 0.001
Monthly income												
Low vs. Medium and High	1.06 (0.65, 1.75)	0.060 0.807	1.23 (0.74, 2.03)	0.628 0.428	1.43 (0.88, 2.32)	2.131 0.144	2.05 (1.21, 3.45)	7.205 0.007	2.30 (1.34, 3.95)	9.107 0.003	4.33 (2.02, 9.27)	14.209 0.000

Probability values (*p*) in bold are significant at a 5% significance level; CI = 95% confidence interval. Prediction Equations: Peanut Butter = 1/(1 + exp(−(0.01 + 0.53 × Age Young + 0.43 × Education Level Post High School))); Peanut Flour = 1/(1 + exp(−(−0.41 + 0.62 × Gender Female + 0.53 × Age Old + 0.53 × Education Level High School and Below))); Roasted Peanuts = 1/(1 + exp(−(0.09 + 0.76 × Gender Male + 0.39 × Occupation Full-time))); Boiled Peanuts = 1/(1 + exp(−(0.69 + 0.44 × Education Level Post High School + 0.72 × Monthly Income Medium and High))); Raw Peanuts = 1/(1 + exp(−(0.49 + 1.34 × Gender Male + 0.78 × Age Old + 1.10 × Education Level Post High school + 0.83 × Monthly Income Low))); Peanut Cooking Oil = 1/(1 + exp(−(−2.20 + 0.94 × Gender Male−0.490 × Age—old + 0.72 × Education Level High School and Below + 0.71 × Occupation Fulltime + 1.47 × Monthly Income Low))).

**Table 3:** Logistic regression estimates for demographic and socioeconomic variables influencing peanut consumption frequency and readiness to try new foods, respectively.

Predictor	Peanut Consumption Frequency		Readiness to Try New Foods	
	Odds Ratio (CI)	χ^2^ *p* > χ^2^	Odds Ratio (CI)	χ^2^ *p* > χ^2^
Gender				
Male vs. Female	1.12 (0.75, 1.65)	0.301 0.583	1.90 (1.27, 2.84)	9.603 0.002
Age				
<30 years vs. ≥30 years	1.01 (0.69, 1.50)	0.005 0.945	1.38 (0.92, 2.06)	2.401 0.121
Education level				
High school and below vs. Post high school	2.35 (1.57, 3.51)	17.301 <0.0001	2.74 (1.79, 4.18)	21.796 <0.0001
Occupation				
Full-time employment vs. Other occupations	1.20 (0.81, 1.77)	0.819 0.366	2.02 (1.35, 3.02)	11.824 0.001
Monthly income				
Low vs. Medium and High	1.16 (0.71, 1.90)	0.337 0.561	1.04 (0.62, 1.75)	0.018 0.893

Probability values (*p*) in bold are significant at 5% significance level; CI = 95% confidence interval. Prediction Equations: Consumption frequency = 1/(1 + exp(−(−0.59 + 0.85 × Education Level High School and Below))); Readiness to Try New Foods = 1/(1 + exp(−(0.40 + 0.64 × Gender Male + 1.01 × Education Level Post High School + 0.71 × Occupation Full-time))).
